# Treatment of Skeletal Class II Division 1 Using Twin Block Myofunctional Appliance

**DOI:** 10.7759/cureus.47713

**Published:** 2023-10-26

**Authors:** Bhakti Kalbande, Vikrant V Jadhav, Amit Reche, Sumukh Nerurkar, Yash Ghulaxe

**Affiliations:** 1 Dentistry, Sharad Pawar Dental College, Datta Meghe Institute of Higher Education and Research, Wardha, IND; 2 Orthodontics and Dentofacial Orthopedics, Sharad Pawar Dental College, Datta Meghe Institute of Higher Education and Research, Wardha, IND; 3 Public Health Dentistry, Sharad Pawar Dental College, Datta Meghe Institute of Higher Education and Research, Wardha, IND; 4 Orthodontics, Sharad Pawar Dental College, Datta Meghe Institute of Higher Education and Research, Wardha, IND; 5 Medicine, Jawaharlal Nehru Medical College, Datta Meghe Institute of Higher Education and Research, Wardha, IND

**Keywords:** malocclusion, mandibular growth, class ii division 1, twin block, myofunctional appliance

## Abstract

Myofunctional appliances are customarily used to treat Class II malocclusions in growing children to alter their growth. Functional appliances are widely accepted to enhance skeletal relationships in the short term efficiently. It utilizes muscular forces by muscles to make dental and skeleton modifications. The myofunctional appliance might be removable or fixed. The variation of mode and method of action depends on the design, but the forces created by the muscles' stretching provide their effect. According to research, the effectiveness of functional appliances as a therapy for Class II malocclusion might be influenced by mandibular growth patterns. Their low skeletal maturation influence outweighs the primary dentoalveolar impact of the twin block myofunctional orthodontic appliances. Class II malocclusions can benefit by using myofunctional appliances in specific clinical situations, such as when the patient is still developing. These devices make the fixed appliance phase easier to use, but their effectiveness depends heavily on the patient's compliance. In this case, an 11-year-old female expressed concern about the forward positioning of her upper front teeth when she visited the department of orthodontics. Twin block, a myofunctional appliance, was used to manage it, and then fixed orthodontic treatment was used to fine-tune the occlusion. This case report illustrates the design and treatment effects.

## Introduction

The dental specialty of orthodontics focuses on improving overbite and straightening teeth. Esthetic procedures have become significantly more popular in recent years [1]. The general public associates success in many endeavors with having attractive teeth. Societal pressures generally set the standards for an acceptable, typical, and appealing physical appearance. Instead of an illness, morphological variation, which may or may not be connected to pathological circumstances, is the fundamental cause of the issue [2]. A malocclusion is an occlusion that deviates from the normal occlusion in one or more of the following ways: the relationship between the maxillary and mandibular arches is aberrant in one or more of the planes, or the location, number, form, or developmental position of the teeth is abnormal [3]. The development of malocclusion can be caused by various etiological factors such as genetics, environmental factors, or a combination of both, as well as several local variables like poor or destructive oral habits [4]. According to the report, 12.5% is the overall prevalence and require orthodontic treatment, and 87.5% did not need a treatment plan. A severe malocclusion that required treatment would be ideal and recommended was recorded in 3.1%; 8% of therapy was elective, depending on the dentist and patient's will [5].

Malocclusion can be categorized in several ways. The most typical malocclusion is Class I, in which the maxillary arch teeth slightly overlap the mandibular arch teeth, and the bite is normal. Retrognathism in the sagittal plane and overbite in the vertical plane is a Class II malocclusion that develops when the upper arch teeth substantially overlap the lower arch teeth. Prognathism in the sagittal plane and underbite in the vertical plane is a Class III malocclusion that develops when the lower jaw puts forward or protrudes, resulting in overlapping of the upper and lower jaws. Maxillary anterior displacement, mandibular retrognathism, increased vertical dimension of the posterior maxilla, position of the mandibular fossa, and several other characteristics are usually associated with Class II malocclusion. In contrast to maxillary incisors, which frequently protrude, upper and lower incisors are frequently well-positioned. Class II skeletal malocclusion is believed to have mandibular retrognathism as its primary underlying cause. Because patients have a wide range of growth patterns and treatment options, Class II division 1 malocclusion is an issue that orthodontists frequently face [6]. Using natural forces, a functional device delivers them in a specified direction to the alveolar bone and teeth. Several intraoral appliances, known as "myofunctional appliances," rely on the orofacial musculature's intrinsic forces to function. These appliances have been used in orthodontics for a long time and often. They are typically passive and detachable. Instead of using active forces, they either transmit, eliminate, or direct the orofacial musculature's inherent forces to repair the dentofacial structures' abnormal growth and function. They are primarily considered for modifying development in Class II division 1 and skeletal Class III disorders [7]. Many functional and orthopedic appliances are available for repairing Class II skeletal and occlusal disharmonies, including Herbst appliances, Bionator 1-3, and fixed FR-2 of Fränkel [8]. One of them was created by William J. Clark in Fife, Scotland, and has amassed a great deal of fame over the previous 10 years. Due to its efficiency and, most crucially, patients' compliance, the twin block myofunctional appliance is frequently utilized in orthodontics. The ideal appliance for treating Class II malocclusions has acrylic mandibular and maxillary plates with bite blocks that move the mandible forward when the mouth is closed. To promote higher growth at the condylar cartilage, the primary objective of mandibular extension is implemented [9]. The main objective for seeking orthodontic intervention in cases of Class II malocclusions is often related to esthetic improvements. Nevertheless, in situations where the malocclusion has a skeletal basis, the available treatment choices may be influenced by the age of the patient [10].

The following presenting case is of an 11-year-old female patient with Class II division 1 malocclusion, and its correction with the help of a twin block myofunctional appliance followed by fixed orthodontic treatment.

## Case presentation

**Figure 1:**
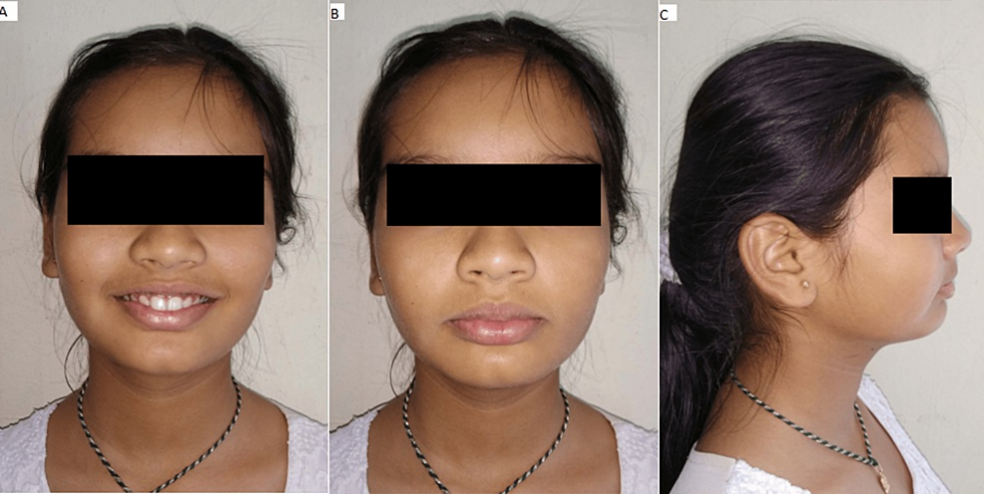
(A) Frontal profile, (B) smiling profile, and (C) side profile

An 11-year-old girl complained to the department that her upper front teeth were positioned too far forward. Upon additional oral examination, a convex profile, mesencephalic head form, average nasolabial angle, and deep mentolabial sulcus were observed (Figure 1).

**Figure 2:**
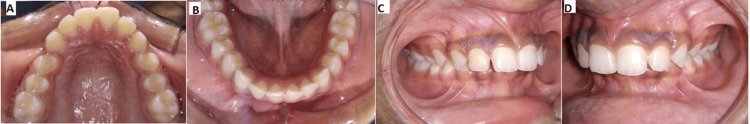
(A) Maxillary arch, (B) mandibular arch, (C) right molar relation, and (D) left molar relation

It showed a class II molar and canine relation, increased overjet, overbite, the deep Spee curve, and minor crowding in the upper and lower anterior on intraoral examination (Figure 2).

**Figure 3:**
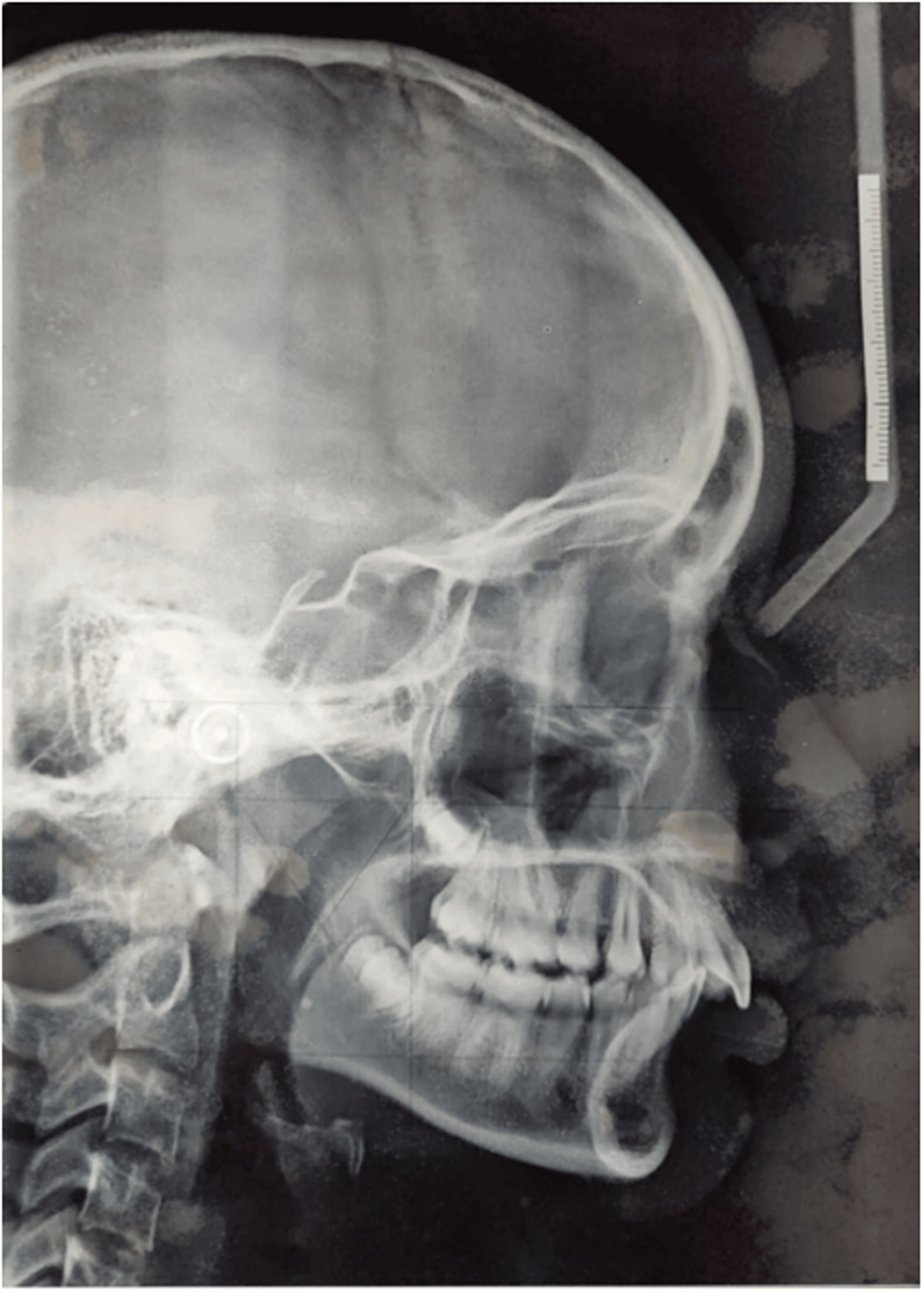
Lateral cephalometric analysis

The cephalometric analysis is shown below (Figure 3).

**Figure 4:**
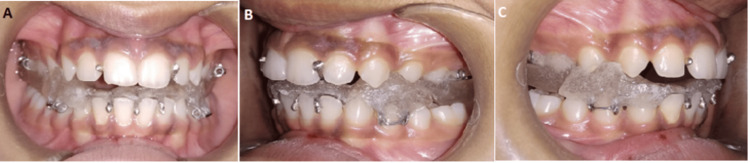
(A) Anterior occlusion, (B) left molar twin block, and (C) right molar twin block

The treatment objective was to attain a Class I canine and molar relation with functional occlusion to achieve a straight profile and have a normal overjet and overbite. The aim also included maintaining the axial inclination of the lateral incisor to increase stability, starting with the appliance for attaining normal overjet and overbite. The appliance used for mandibular advancement was the Clark's twin block. This modified version of the twin block design in terms of sagittal advancement and vertical opening not only targets Class II malocclusions but also effectively manages transverse discrepancy. Ideally, a 6:4 ratio is followed for the fabrication of a twin block appliance in which 6 mm is sagittal advancement and 4 mm is vertical opening. The appliance is designed to create changes in the bite in both the sagittal and vertical dimensions, featuring a 7 mm sagittal advancement and a 5 mm vertical opening. Additional delta clasps are added in the maxillary arch replacing the traditional labial bow concept. Moreover, the concept of anterior labial covering is replaced with an additional delta clasp. The pictures below show the placement of a twin block appliance in anterior occlusion on the side of the right molar and left molar (Figure 4).

**Figure 5:**
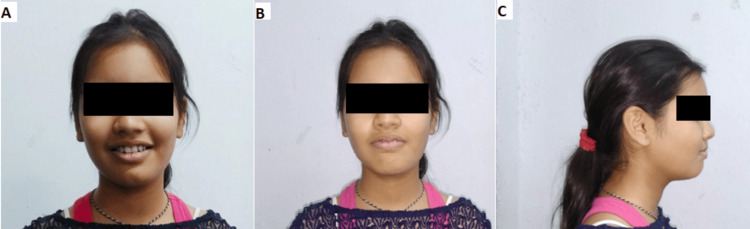
(A) Smiling profile, (B) frontal profile, and (C) side profile

The facial profiles, i.e., smiling, frontal, and side profiles, of the patient during mid-treatment are shown below (Figure 5).

**Figure 6:**
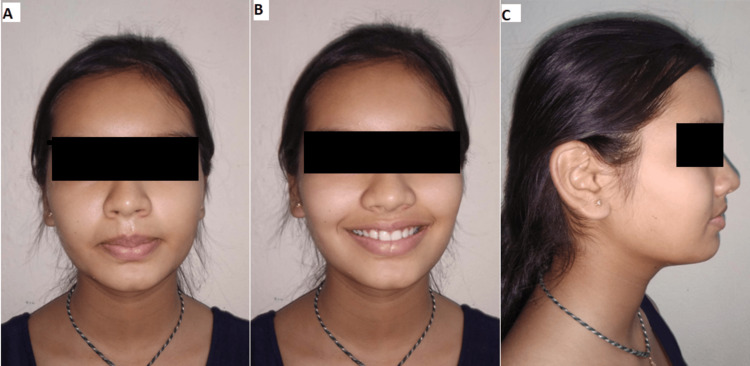
(A) Frontal profile, (B) side profile, and (C) smiling profile

These resulted in achieving a Class I molar and canine relation (Figure 6), showing pictures after myofunctional therapy.

**Figure 7:**
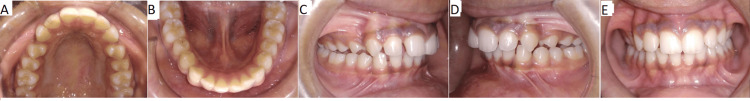
(A) Maxillary arch, (B) mandibular arch, (C) right molar relation, (D) left molar relation, and (E) anterior view

Intraoral photographs of the patient after the use of myofunctional therapy are shown below (Figure 7).

**Figure 8:**
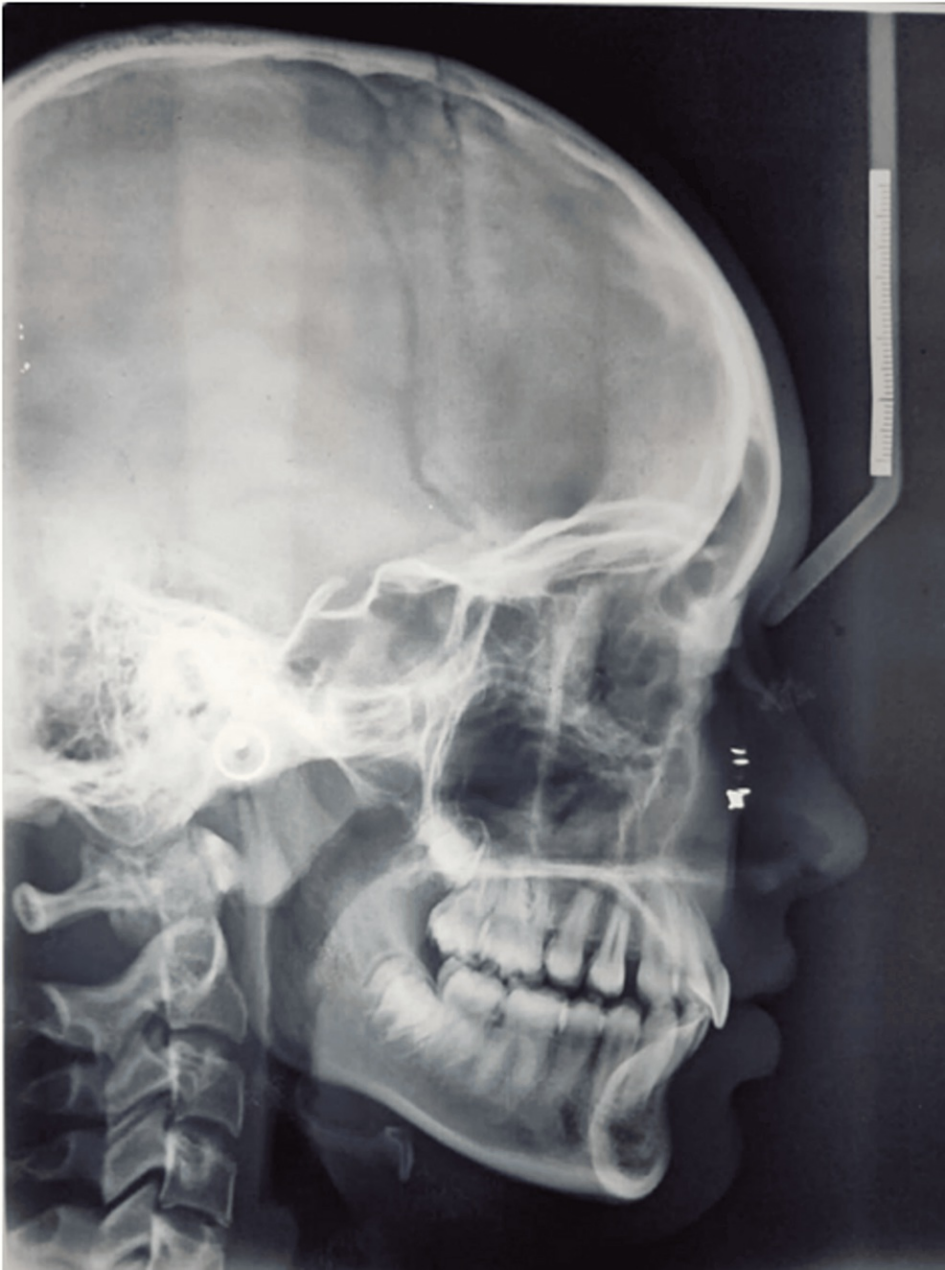
Lateral cephalogram

The lateral cephalogram is shown below (Figure 8).

**Figure 9:**
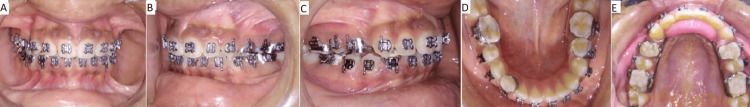
(A) Anterior view, (B), left molar relation, (C) right molar relation, (D) mandibular arch, and (E) maxillary arch

Since minor tooth movements and finishing are not possible with the help of a functional appliance, the lower and upper arches were bonded using 0.022” slot MBT brackets. The initial leveling and alignment were done using the following wire sequence: 0.016˝ nickel-titanium (NiTi), followed by 0.016 and 0.022˝ NiTi, 0.17 and 0.025˝ NiTi, and finally 0.19 and 0.025 stainless steel (SS). The changes achieved by the twin block were retained with the placement of a fixed anterior inclined plane, as shown below (Figure 9).

Treatment result

**Figure 10:**
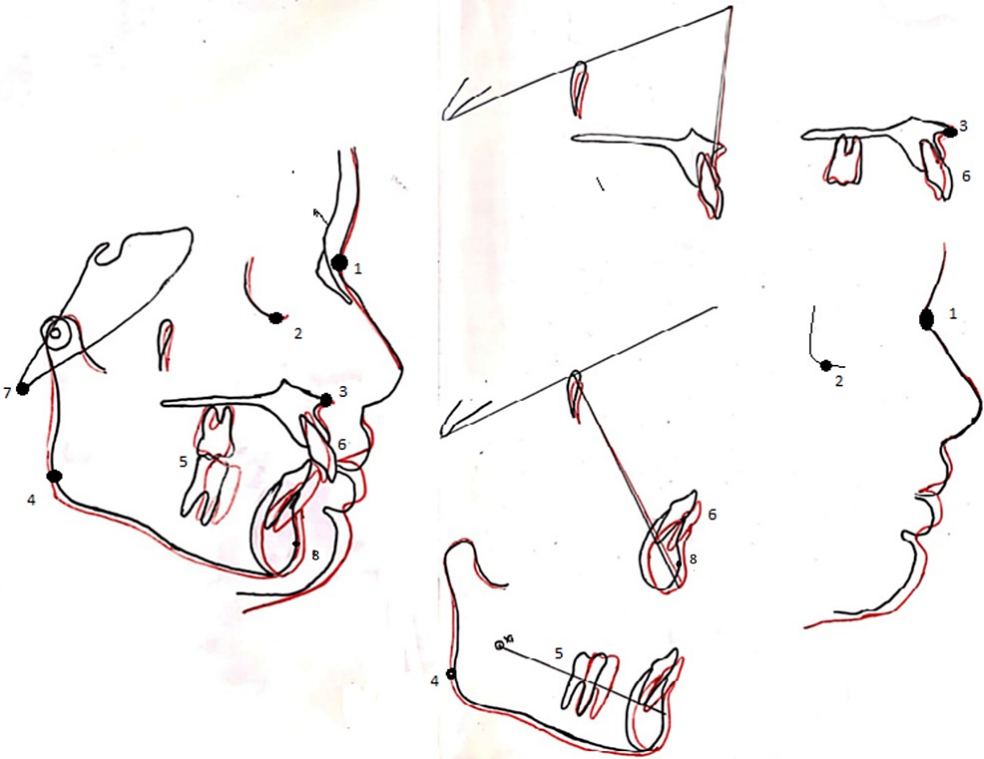
Superimposition of the maxilla and mandible The black line indicates pretreatment and the red line indicates post-treatment. 1. Nasion; (2) orbitale; (3) anterior nasal spine; (4) gonion; (5) molar; (6) incisor; (7) basion; (8) pogonion.

The treatment's goals were accomplished. Following the therapy, the patient's profile improved. The proclination of the lower incisors alleviated the lower arch crowding. During the phase of treatment where a fixed appliance was used, the spacing of the upper arch was closed using an e-chain. By completing the therapy, the molar, canine, and incisor connections were class I. The average was applied to the overbite and overjet. The illustration shows the entire superimposition of the maxilla and mandible (Figure 10). These images reveal the growth changes; black indicates pretreatment, and red signifies post-treatment.

## Discussion

Based on the patient's age, growth, and skeletal maturity, there are several ways to treat Class II division 1 malocclusion. The most common treatment approach is functional appliance therapy [11]. Dr. William Clark developed the twin block, a two-piece device designed to correct the mandible's occlusion relation. The primary advantage of the twin block appliance over other functional appliances is that it can be worn 24 hours a day, and the masticatory force assists in repositioning the mandible forward. After the mandibular advancement is complete, an anterior inclined plane is placed for retention, and it is retained until the posterior occlusion stabilizes. Twin block functional appliances offer several well-known benefits, are well-received by the patients, and can be used in both permanent and mixed dentition. They are also easy to maintain. However, two potential drawbacks of the twin block appliance are the advancement of a posterior open bite and the proclination of the mandibular incisors. In this case, the patient's excellent compliance was crucial to achieving therapeutic goals, and the patient's confidence has grown due to the functional appliance's ability to reduce overjet and the decreased risk of trauma to the upper incisors [12]. The Delta clasp, developed by Clarks in 1985, was introduced to enhance the fixation of twin block appliances. The Adams clasp served as a precursor to the Delta clasp, and its name is derived from the shape of its retentive part. It is commonly used with the Clark twin block appliance, where occlusal interferences are not a concern, and a clasp with a high retention level is required [13]. In 1973, Harvold described histological changes related to this type of treatment [14], while in 1980, McNamara reported immediate changes in neuromuscular proprioceptive response [15]. Additionally, acrylic was added to the lower labial bow of the twin block to enhance retention, and the appliance also includes a ball clasp for retention on the lower arch. If lateral arch development is necessary, expansion screws can be placed in either the upper or lower arch.

The twin block appliance, commonly used in orthodontics to correct Class II malocclusions, comes in various modifications and adaptations to provide specific patient needs and treatment objectives. Here, several distinct variations of the twin block appliance are discussed below (Table 1). Each of these modifications has distinct indications and is employed by orthodontists according to individual patient needs and treatment objectives. Patients should consult their orthodontist to determine the most appropriate appliance for their specific case.

**Table 1:** Various modifications of the twin block appliance [16-20].

Appliance name	Description
Twin block appliance for transverse and sagittal development	Targets Class II malocclusions, addressing transverse and sagittal developmental concerns in jaw alignment.
Twin block Croat appliance	A specialized adaptation of the standard twin block, featuring unique features or enhancements developed by orthodontist Croat.
Magnetic twin block	Incorporates magnetic components to improve the mechanics and effectiveness of the twin block appliance.
Twin block with spinner and occlusion rest	Includes a spinner mechanism for greater customization and adaptability during the treatment process.
Fixed twin block	Permanently affixed to the patient's teeth, primarily used when patient compliance with a removable appliance is a concern.
Reverse twin block	Specialized for Class III malocclusions, encouraging forward growth of the mandible.
Twin block hybrid appliance	Combines aspects of the twin block with other orthodontic devices, offering solutions for complex cases.
Neuromuscular twin block (Gerber Banded Block)	Designed with a focus on neuromuscular considerations to optimize the function of jaw muscles during treatment.
Twin block for Class II Div 2	Specifically created for patients with Class II division 2 malocclusions, characterized by a pronounced overbite.
Twin block appliance with bite-jumping screw for progressive advancement	Incorporates a bite-jumping screw for controlled and gradual advancement of the lower jaw.
Implant-supported twin block	In select cases, implants provide support and stability to the twin block appliance, especially when natural tooth support is inadequate.

## Conclusions

The twin block is a full-time wear appliance that corrects the maxillomandibular relationship by promoting functional repositioning of the mandible. It modifies the occlusal inclined plane and guides the mandible forward into the correct occlusion. The upper and lower bite blocks interlock at a 70° angle. Twin block functional appliances primarily have dentoalveolar effects with minor skeletal components. They benefit from the functional forces acting on the dentition, which simplifies the subsequent stage of fixed appliance orthodontics. In the discussed case, an 11-year-old patient was treated with a twin block appliance before the fixed appliance orthodontic phase. This case study illustrates the influence of the appliance's design.
